# Potential corridors and barriers for plague spread in central Asia

**DOI:** 10.1186/1476-072X-12-49

**Published:** 2013-10-31

**Authors:** Liesbeth I Wilschut, Elisabeth A Addink, Hans Heesterbeek, Lise Heier, Anne Laudisoit, Mike Begon, Stephen Davis, Vladimir M Dubyanskiy, Leonid A Burdelov, Steven M de Jong

**Affiliations:** 1 Department of Physical Geography, Utrecht University, Heidelberglaan 2, PO Box 80115, Utrecht 3508 TC, The Netherlands; 2 Faculty of Veterinary Medicine, Utrecht University, Yalelaan 7, Utrecht 3584 CL, The Netherlands; 3 Department of Biosciences, University of Oslo, PO Box 1066 Blindern, Oslo NO-0316, Norway; 4 Ecology Evolution and Genomics of Infectious Disease Research Group, Institute of Integrative Biology, The University of Liverpool, Liverpool, UK; 5 Department of Biology, University of Antwerp, Groenenborgerlaan 171, Antwerpen B-2020, Belgium; 6 School of Mathematical and Geospatial Sciences, RMIT University, Melbourne, Victoria 3000, Australia; 7 M. Aikimbayev's Kazakh Science Centre for Quarantine and Zoonotic Diseases, 14 Kapalskaya Street, Almaty 050074, Kazakhstan; 8 Stavropol Plague Control Research Institute, Sovetskaya 13-15, Stavropol 355035, Russian Federation

**Keywords:** Landscape epidemiology, Disease ecology, Infectious disease, Remote sensing, Persistence, GIS, Multiple regression, Landscape configuration, Steppe, Bubonic plague

## Abstract

**Background:**

Plague (*Yersinia pestis* infection) is a vector-borne disease which caused millions of human deaths in the Middle Ages. The hosts of plague are mostly rodents, and the disease is spread by the fleas that feed on them. Currently, the disease still circulates amongst sylvatic rodent populations all over the world, including great gerbil (*Rhombomys opimus*) populations in Central Asia. Great gerbils are social desert rodents that live in family groups in burrows, which are visible on satellite images. In great gerbil populations an abundance threshold exists, above which plague can spread causing epizootics. The spatial distribution of the host species is thought to influence the plague dynamics, such as the direction of plague spread, however no detailed analysis exists on the possible functional or structural corridors and barriers that are present in this population and landscape. This study aims to fill that gap.

**Methods:**

Three 20 by 20 km areas with known great gerbil burrow distributions were used to analyse the spatial distribution of the burrows. Object-based image analysis was used to map the landscape at several scales, and was linked to the burrow maps. A novel object-based method was developed – the mean neighbour absolute burrow density difference (MNABDD) – to identify the optimal scale and evaluate the efficacy of using landscape objects as opposed to square cells. Multiple regression using raster maps was used to identify the landscape-ecological variables that explain burrow density best. Functional corridors and barriers were mapped using burrow density thresholds. Cumulative resistance of the burrow distribution to potential disease spread was evaluated using cost distance analysis. A 46-year plague surveillance dataset was used to evaluate whether plague spread was radially symmetric.

**Results:**

The burrow distribution was found to be non-random and negatively correlated with Greenness, especially in the floodplain areas. Corridors and barriers showed a mostly NWSE alignment, suggesting easier spreading along this axis. This was confirmed by the analysis of the plague data.

**Conclusions:**

Plague spread had a predominantly NWSE direction, which is likely due to the NWSE alignment of corridors and barriers in the burrow distribution and the landscape. This finding may improve predictions of plague in the future and emphasizes the importance of including landscape analysis in wildlife disease studies.

## Introduction

Spread of pathogens through a population of hosts can be enhanced or restricted by the landscape. The landscape offers a ‘matrix’ of more and less suitable habitat [1] and therefore can influence both the abundance and movements of hosts and vectors [2-4]. Suitable habitat areas for hosts and/or vectors may lead to a higher contact rate of hosts [5] and vectors, and these areas therefore may act as corridors for pathogen transmission through a larger landscape. On the other hand, unsuitable habitat may prevent transmission because it cannot be crossed by hosts or vectors, and thus such areas may act as barriers for pathogen transmission [6]. Corridors and barriers can be present at various spatial scales, depending on the movement and migration patterns of the host and vector. The influence of the landscape or the spatial distribution of the host or vector population on infectious disease dynamics has been shown for several diseases, such as those caused by hantavirus [7,8], Lyme disease [9] and plague [10-12]. In this paper, we focus on plague.

Plague is a vector-borne zoonotic disease that is caused by the bacterium *Yersinia pestis*. The bacterium has a broad host range, but mostly infects rodents [13], and is spread from host to host by fleas. In Eastern Kazakhstan, the presence of this pathogen has been monitored in great gerbils (*Rhombomys opimus*), social rodents that live in burrows, for decades. It has been found that outbreaks of plague occur only when a host and flea abundance threshold is exceeded [14,15], but the direct influence of the landscape on spatial spread and the dynamics of this disease has not previously been investigated.

This study investigates the role of the landscape and the host distribution on the occurrence of plague in the PreBalkhash focus in Eastern Kazakhstan. In this area, the burrows of the great gerbil were recently mapped with considerable success across several landscape types using semi-automated object-based classification [16]. These burrow maps will be used to analyse burrow distribution patterns as a proxy for patterns in a network of great gerbil contacts for the spread of plague. The landscape will be analyzed using characteristics derived from satellite images and a digital elevation model.

We distinguish *functional* and *structural* corridors and barriers. *Functional barriers* are areas where, due to the spatial distribution of hosts, a disease is expected to spread with greater difficulty or not at all. In the PreBalkhash focus, these are areas where relatively few or no great gerbil burrows are present. *Functional corridors* are areas where, due to the spatial distribution of hosts, a disease is expected to spread more easily. In the PreBalkhash focus, these are areas where the great gerbil burrow density is relatively high. *Structural barriers*, then, are landscape features that are difficult or impossible for great gerbils to cross. *Structural corridors* are not identified in this study, but are defined as landscape features where movement of individuals is enhanced (such as roads in cases where humans are the transmitting hosts).

In order to gain insight into the role of the landscape in the occurrence of plague in Eastern Kazakhstan, we ask the following questions based on the burrow distribution data:

1. Is the distribution of burrows random, regular or aggregated? At what scale is any structure most pronounced?

2. Which landscape-ecological variables best explain burrow density?

3. Can we identify corridors and barriers in the burrow distribution and landscape? If so, what is their spatial configuration and how will this potentially influence the direction and speed of plague spread through the landscape, assuming burrow densities correlate with the speed of plague spread?

And we ask the following question based on the epidemiological data:

4. Using 46 years of historical epidemiological data, was the observed spread of plague radially symmetric?

### Background and study area

#### The plague system

**Figure 1:**
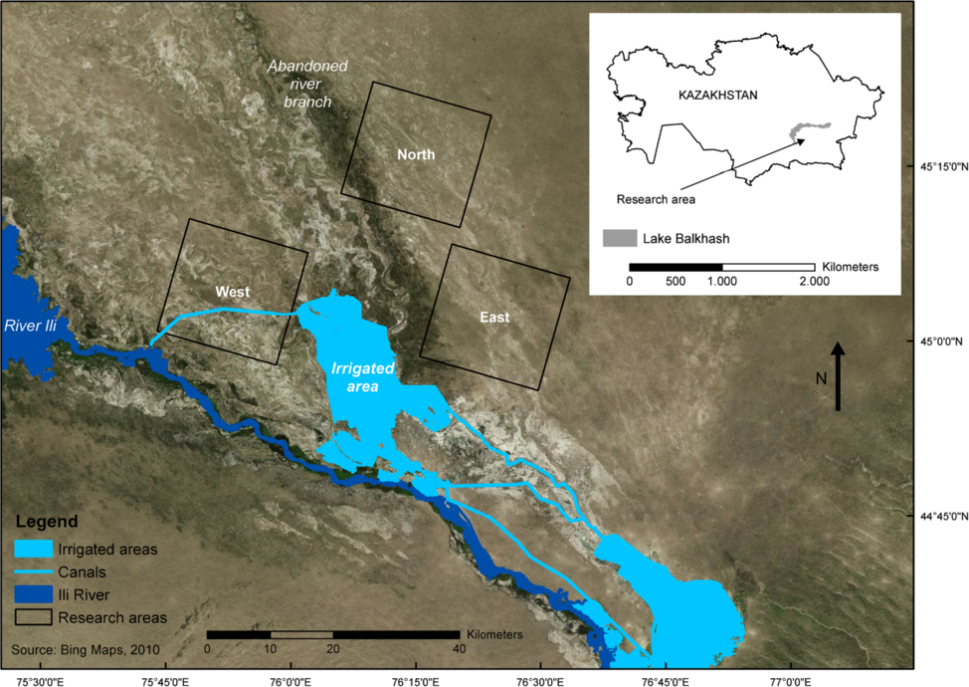
**The plague focus with the research areas *West*, *North *and *East*.** The irrigated areas, canals and the Ili River are shown in blue. The inset shows the location of the study area in Kazakhstan, south of Lake Balkhash.

The study area is located in the Balkhash basin in Eastern Kazakhstan, north of the Ili River (Figure 1), though plague occurs both north and south of the river. It is classified as a plague focus, meaning that hosts, vectors and plague bacteria are present. The primary host in this focus is the great gerbil [17], which is distributed across large areas of Central Asia.

Great gerbils live in family groups usually consisting of one male, one or more females and their offspring [18]. While foraging, great gerbils usually stay close to their burrow entrances [19]. When gerbils disperse, defined as gerbils permanently moving from their natal colony to another colony during the first year of their life, they move larger distances: dispersal distances for males and females are on average 350 m and 150 m, respectively [18].

The main vectors of *Yersinia pestis* are fleas of the genus *Xenopsylla*[20]. The abundance of the fleas varies with an approximately two-year delay with great gerbil population fluctuations. In addition, flea abundance is influenced by the microclimate inside burrows, which is influenced by the local weather conditions. For example, flea survival decreases when temperatures are high and air humidity is low [20].

#### The landscape

Like many current plague foci, the PreBalkhash focus is located in a semiarid area [21]. At the landscape level, the study area (Figure 1) can be described by three main landforms: the active river, floodplains and dunes. Overall, vegetation cover is low and has a decreasing trend towards Lake Balkhash. Soils are sandy, with variable clay and low organic matter content.

**Figure 2:**
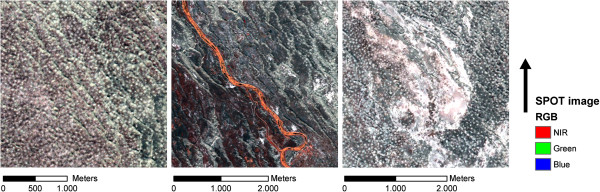
**Examples of local variations in the landscape.***Left*: dunes with burrows. *Middle*: an abandoned river branch with green vegetation, *Right*: a takir surrounded by burrows.

At a more local level, variations in environmental conditions exist, because, amongst other reasons, precipitation is sporadic and spatially localized. In the floodplain areas, quick snow melt in spring results in lakes (called takirs) that form in depressions [22]. In clay-rich areas larger takirs (Figure 2) develop when settling of the soil occurs. These takirs may act as a barrier to the great gerbils, as it is difficult for them to construct burrows into these hard and consolidated soils. Furthermore, the takirs have very little vegetation cover, which does not make them attractive for foraging.

The vegetation cover on the floodplains ranges from totally bare to abundant shrub vegetation. Vegetation cover is higher near to abandoned river branches (Figure 2), most likely due to subsurface flow and hence higher moisture availability. In the dunes, variations in vegetation cover are most pronounced. Vegetation ranges from very sparse short grass vegetation, to more abundant long grass and small shrub vegetation.

#### The burrows of the great gerbil

The burrows of the great gerbil are omnipresent in the landscape (Figure 2). On the surface of a burrow that is presently or has recently been occupied, no or relatively little vegetation is present, because this has been removed by the great gerbils. Burrows are typically round in relatively flat areas and more elongated in the dunes and have a diameter of 20-60 m [16]. Their large size and bare surface enable the recognition of burrows on satellite images. Burrows have multiple entrances that lead to a network of subterranean tunnels and chambers. Depending on the stability of the soil, a burrow can extend to three meters in depth [23]. Deeper burrows will have a more stable temperature inside the chambers and hence are likely to increase survival of the gerbils [24]. Shallow burrows may provide a barrier to fleas, as fleas are less prone to survive extreme temperatures and low humidity [20].

### Data

#### Satellite images

Landsat 7 ETM + images (28.5 m resolution) from 2000 and 2001 were used for the creation of multi-scale landscape objects, together with a 90 m resolution Digital Elevation Model (DEM) acquired by the Shuttle Radar Topography Mission (SRTM). Landsat images were converted to top-of-atmosphere reflectance [25].

#### Burrow maps

In a previous study, burrows of the great gerbil were mapped in two areas covered by high-resolution satellite images [16]. The 2.5 m pixels in the images were grouped into objects, at a scale such that they coincided with the burrows. The burrow objects were then separated from non-burrow objects by a Random Forest classifier, using spectral and neighbour signatures. The end product of that study has a mean overall accuracy of 90%. For the present study, the polygon-objects representing burrow systems were converted into point data files, using the coordinates of the centroid of the polygon as burrow locations.

#### Plague data

The plague dataset consists of biannual data collected by the Kazakh Scientific Center for Quarantine and Zoonotic Disease (formerly Anti-Plague Institute) between 1949 and 1995 in about 90 so-called Primary Squares (PSQs). PSQs are plague monitoring units (measuring 18.5 km by 19.6 km) that were and are still used by the anti-plague services in Kazakhstan. Every spring and autumn great gerbils and their fleas were caught in a number of PSQs and tested for infection with *Yersinia pestis*. The plague dataset thus shows presence or absence of plague at different locations in the PreBalkhash focus through the years. For more detailed information on the collection of the data set we refer to a previous study [26].

In total, the data set used consists of 3493 data points, distributed over the 90 PSQs. Of those data points, 1750 were collected in spring and 1743 in autumn. In 8.8% of the data points, plague was detected.

## Methods

### Selecting research areas

To analyse the burrow distribution and identify corridors and barriers, three research areas were selected. The size of the research areas was chosen to match the size of a PSQ, because this may simplify future comparisons with plague data. The areas were selected so that most dominant natural landscape types in the area were covered, and urban and irrigated areas were avoided. In addition, the research areas were selected so that both dunes and floodplains occurred in different fractions (Table 1). Research area *West* consists almost completely of floodplain. Research area *North* has an even distribution of both dunes and floodplain, and research area *East* consists of 86% floodplain and 14% dunes.

**Table 1:** Cover of floodplain and dunes in the three research areas

	**Floodplain (%)**	**Dunes (%)**
*West*	99	1
*North*	53	47
*East*	86	14

### Characterising burrow patterns

To characterise the burrow distribution patterns, nearest neighbourhood statistics were calculated for the burrow patterns in each research area using the statistical environment R [27]. Also, variograms [28] were created to get insight in the spatial correlation in the abundance of burrows, by first rasterizing the burrow maps into burrow-density maps, using cells of 100 m, 400 m and 1000 m, respectively. The range was also calculated for each variogram, using the R package gstat.

For each research area, 100 random dummy burrow patterns with an equal number of burrows as in the real burrow pattern were generated using the R package spatstat, to be able to compare completely random spatial point patterns with the burrow distributions.

### Analysing the spatial burrow density pattern using landscape objects

To investigate whether there is a spatial structure in the burrow distribution and whether there is a spatial scale at which the spatial structure is most pronounced, the density of burrows across the landscape was evaluated using multi-scale object-based image analysis (MSOBIA). MSOBIA has been suggested for analysing the relationship between landscape and animal abundance [29,30], but, at least until now, has never been used with real animal abundance and distribution data. In the present study, the burrow maps make it possible to test whether landscape objects coincide with the patterns in the burrow maps.

#### Creating landscape object layers

The MSOBIA was performed using Landsat ETM + satellite images and an SRTM elevation model. Three layers were extracted from the images: Tasselled Cap [31] soil Brightness (Brightness), Tasselled Cap Greenness (Greenness), and the standard deviation in the SRTM elevation (SRTM-SD). Brightness is a proxy for soil colour and hence soil type, Greenness is a proxy for the vegetation cover, and SRTM-SD is a proxy for the variation in the local topography. Together they capture the major variation in the landscape.

The three layers were segmented into objects representing the landscape patches by grouping neighbouring pixels based on a homogeneity criterion. At a high homogeneity criterion value, i.e. the pixels are required to be quite similar in order to be grouped, many small objects are created, whereas at a low homogeneity criterion value, larger objects are obtained. Segmentation was started at low homogeneity with a mean object size of ~17 km^2^, and was then repeated step-wise with increasing homogeneity. In the final layer with most objects, the mean object size was approximately 200 m^2^. In total 20 landscape object layers were produced. Segmentation level was plotted against mean object area for each research area, to compare the landscape homogeneity in the three areas. Also, mean values for Greenness, Brightness and SRTM-SD were calculated.

#### Calculating burrow density differences within landscape objects layers

If the landscape objects coincide with the burrow maps, neighbouring objects should show differences in burrow density. To test whether this is the case, and whether the patterns in burrow density are different from random patterns, we developed the metric *mean neighbour absolute burrow density difference* (MNABDD):

$$\mathrm{MNABDD}=\frac{1}{m}\sum_{i}{}^{m}(\frac{1}{n}\sum^{n}{}_{j=n}\left(\left|BD_{i}-BD_{\mathrm{ij}}\right|\right)$$

Where

m = number of objects

n = number of neighbouring objects to object i

BD_i_ = burrow density of object i

BD_ij_ = burrow density of jth object neigbouring object i.

This variable will show relatively high values when landscape objects coincide with burrow density patterns, and low values when they do not coincide. Also, when the burrow pattern itself is random, instead of spatially structured, the value of MNABDD will be low. MNABDD was calculated as follows. First, for all the objects in the landscape object layers a burrow density value was calculated. To prevent extreme density values, objects at the edge of the research areas smaller than 0.025 km^2^ were removed.

The landscape object layers with their associated burrow density values were then used to calculate, for every object, the absolute neighbour burrow density difference (NABDD), i.e. the difference in burrow density between an object and its neighbouring objects. After this, MNABDD was calculated by taking the mean of the NABDDs for every layer.

**Figure 3:**
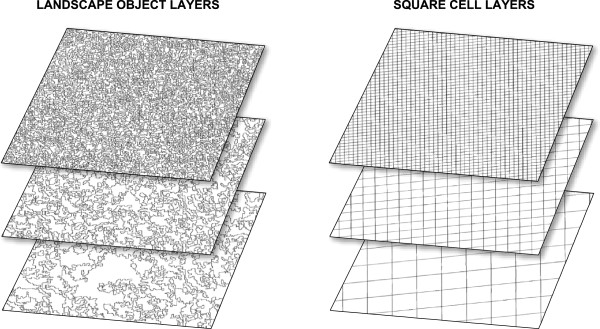
**Example of three landscape objects layers and three square cell layers.** In total 20 landscape object layers and 20 square cell layers were used for the analysis. For each of these layers the MNABDD was calculated.

To evaluate the effect of the landscape objects on MNABDD, MNABDD was also calculated using layers with square cells (Figure 3). If the landscape objects explain variations in burrow density well, then the MNABDD will be higher compared to a MNABDD value calculated from equal sized cells that are unrelated to the landscape. These 20 square cell layers were created by matching the size of the cells in each layer with the mean object size from the 20 landscape object layers for each of the three study sites.

Finally, to evaluate whether the burrow patterns in the three research areas are spatially structured rather than randomly distributed, MNABDD was calculated using the 100 random dummy point patterns. For every research area, using the landscape object layers and the square cell layers, the confidence intervals of MNABDD using the 100 random point patterns were calculated.

### Relating burrow density to landscape-ecological variables

To investigate which landscape-ecological variables best explain burrow density, multiple linear regression was performed. Ideally, in cases where burrow density can be explained very well using the landscape-ecological variables, the model can be used to predict burrow density for areas where no burrow maps are available. This could be very helpful for epidemiological models.

Multiple linear regression was performed using the values from the rasterized burrow maps with a cell size that was well within the variogram range, and also small enough to capture local variations in burrow density. Four explanatory variables were used: Greenness, Brightness, SRTM-SD and SRTM. Second order interactions were also included. Spatial autocorrelation was accounted for in the model [32]; that is, the model fitted to data was a linear mixed-effects model with an exponential correlation structure with a nugget effect (function lme, in R package nlme). We used the AIC [33], a measure of the relative quality of a model, to determine the relative support for each model. This was first done for the three research areas combined. The model with the lowest value of AIC was chosen in case ΔAIC was greater than 2. A similar model using the same combination of variables was then also applied for the three areas separately, so that differences between the three areas could be evaluated.

### Mapping corridors and barriers

To detect the functional corridors and barriers in the burrow distribution, burrow densities were investigated using the landscape object layers. To determine thresholds for the corridors and barriers, the mean and standard deviation of the burrow densities in the three research areas (using the 400 m rasters) were extracted. The threshold for the functional corridors was set at mean burrow density plus one standard deviation. The threshold for the functional barriers was set at mean burrow density minus one standard deviation.

Then the functional corridors and barriers were mapped in each research area, using only areas larger than ~700 m × 700 m (twice the average great gerbil migration distance [18]).

The structural barriers were mapped by identifying the rivers, canals and irrigated agricultural areas in the area. This is because great gerbils do not likely cross them [34], and the infected fleas on them are inclined to leave the gerbil when they go into the water.

#### Analysing the spatial configuration of functional corridors and barriers

**Figure 4:**
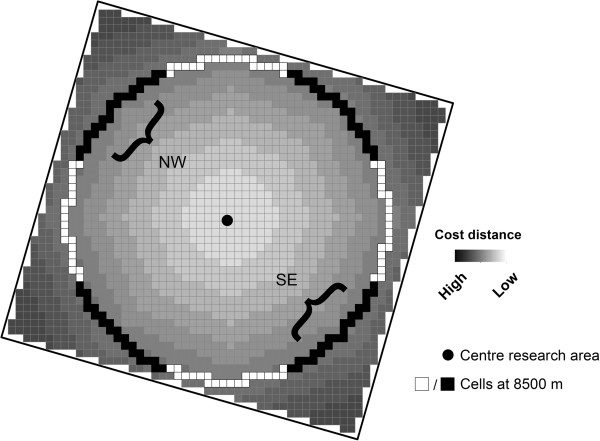
**An example of cost distance.** The black and white cells give the location where the cost distance values are extracted. As an example the cells for the calculation of the NWSE axis are indicated.

To quantify how the distribution of functional corridors and barriers potentially influences the direction of plague spread across the landscape, the *cumulative resistance* of the burrow distribution in different directions was calculated. It was (again) assumed that plague will spread more easily if burrow densities are high, and plague will spread less easily if burrow densities are low. As a measure for cumulative resistance, the cost distance [35] for plague to travel from the sides of the research areas to the centre was calculated based on a resistance map. The cost distance can simply be defined by the accumulated ‘cost’ or resistance it requires to migrate from one place to another. The cost distance was calculated using a resistance map as input, which was calculated by taking the inverse of the 400 m burrow density rasters, so that high burrow densities – the corridors - result in a low resistance and low burrow densities – the barriers - result in a high resistance. Next, the cost distance was calculated for all raster cells at a distance of 8500 m of the centre. The results were then summarized by the four main axes NS, WE, NWSE and NESW for the three areas (Figure 4), so that could be quantified in which direction plague spread potentially could occur most easily.

The procedure was repeated using a homogeneous burrow density map as input, with values equal to the mean burrow density of the three research areas. The resulting mean cost distance value was used to normalize the cumulative resistance axis.

### Analysing the direction of plague spread in the plague data set (1949–1995)

If all conditions were equal in all directions, i.e. if great gerbil densities and abundances were equal and no structural barriers or corridors existed, plague spread can be assumed to have been on average radially symmetric. The spread of plague might however be influenced (in) directly by the landscape.

**Figure 5:**
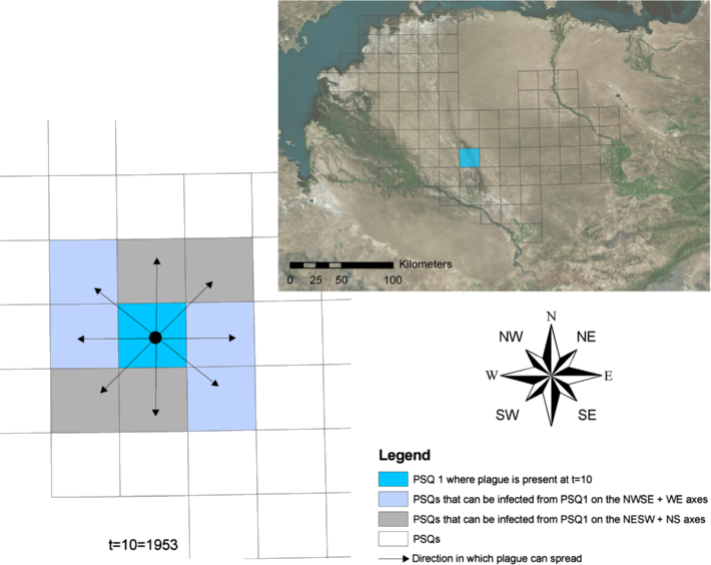
***(Left) *When plague is present in a PSQ (blue square) at for example *t*** **= 10, it may spread to one or more of the eight neighbouring PSQs (shown with arrows).** In the analysis, spread on the axes NWSE and WE (violet squares) was combined, and contrasted with spread on the perpendicular axes, NESW and NS (grey squares). An analysis with the other possible combination, i.e. NWSE + NS vs. NESW + WE, was also performed. The inset on the right shows all PSQs used in the model.

To investigate whether plague spread in the focus in the years 1949–1995 had a predominant direction along one of the four axes (NS, WE, NWSE, NESW), an adapted version of the model of Heier et al. [26] was used (Additional file 1). The adapted model includes variables describing directional spread rather than one variable describing spread in unspecified directions. This was necessary as a simple tabulation of plague observations would not give reliable results; firstly because the sample sizes were often small so that plague, if it was present at a low prevalence, in many cases may not have been found. Secondly, spread depends strongly on gerbil abundance, which may produce noise or bias in the results if not accounted for. In the model of Heier et al. these factors were included. The PSQs that were used for this analysis are shown in Figure 5.

As several gaps in the data exist, the variables describing directional spread were aggregated two by two. In effect, two nearly identical models were fitted to the data: one with the two variables describing NWSE + WE (*ax*1) and the perpendicular NESW + NS (*ax*2); and one with variables describing the other two axes. The two models predict the probability that plague will be present in a given PSQ *i* at time *t* (*y*_*i*,*t*_ = 1) given four explanatory variables: the presence/absence of plague in the same square six months earlier (*y*_*i*,*t*-1_); the proportion of neighbouring squares on one of the four combined axes where plague was present six months earlier (*n*^*ax*1^_*i*,*t*-1_); the proportion of neighbouring squares on the perpendicular axis where plague was present six months earlier (*n*^*ax*2^_*i*,*t*-1_); and the great gerbil abundance in the square, averaged over the past two years (*g*_*t,i*_). The models were used to test whether plague spread was significantly stronger along the NWSE + NS, NESW + NS, NWSE + WE or NESW + WE axes (see spatial example in Figure 5). Estimates of the magnitude of spread along these axes were also obtained.

## Results

### Burrow network characteristics

**Figure 6:**
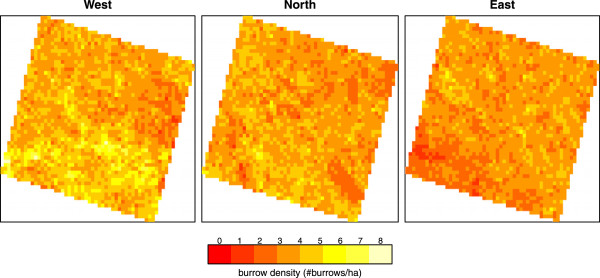
Burrow densities (burrows/ha) per research area using 400 by 400 m cells.

In the three areas *West*, *North* and *East*, 146,101, 132,517 and 123,886 burrows were mapped, respectively. Mean densities (Figure 6) were 4.0, 3.6 and 3.4 burrows per hectare, and mean nearest neighbour distances 30 m, 32 m, and 36 m, respectively. All three research areas thus show a dispersed pattern, i.e. the mean nearest neighbour distances are significantly larger than the complete spatial random point patterns with the same number of burrows (Student’s t-test with unequal variances, p-values = *West*: 2.2e-16, *North*: 1.204e-08, and *East*: 2.2e-16).

The variograms (Additional file 2: Figure S1) show that variation in burrow density is highest in area *West* and lowest in area *East*. Furthermore, they show that the range, or in other words the length at which there is spatial structure in the data, varies from ~2.4 km in area *North* and ~3 km in area *East*, to ~5.2 km in area *West*.

### Landscape characteristics

**Figure 7:**
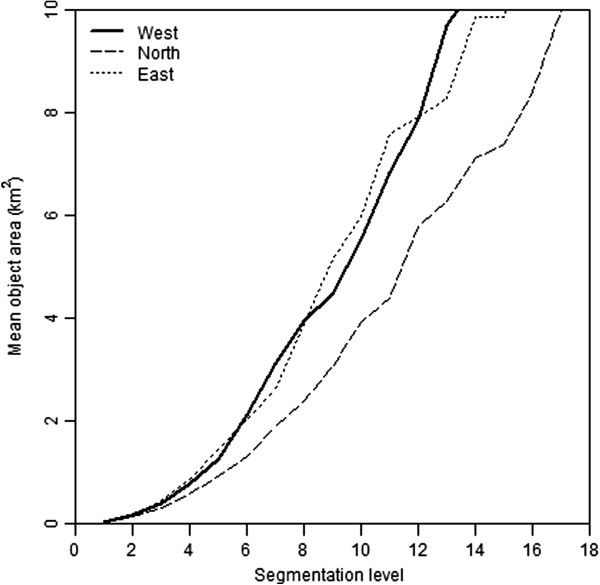
Relation segmentation level (based on a homogeneity threshold) and mean object size for the three research areas.

Landscape segmentation was carried out for the three research areas and a plot of segmentation level versus mean object area (Figure 7) shows that *North* has the smallest objects at all scales and can therefore be classified as the most heterogeneous landscape of the three areas. The greenest of the areas is *East*. Most variation in local topography occurs in area *North*, whereas area *West* is the flattest.

### Burrow density distribution at increasing scales

**Figure 8:**
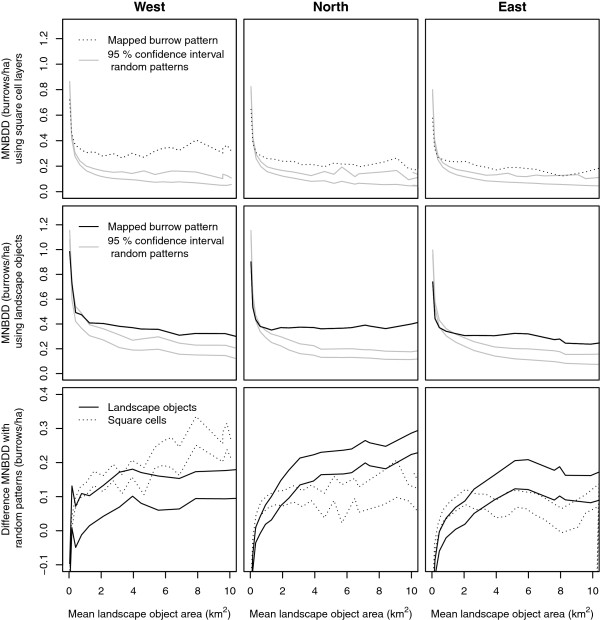
**Mean differences in burrow density between neighbouring objects, for the three research areas.** Top row: square cell layers. Middle row: landscape objects. Bottom row: difference between burrow patterns and random patterns.

The mean neighbour burrow density difference (MNABDD) was calculated for the three research areas (Figure 8). The top row in Figure 8 shows the MNABDD values for the square cell layers; the middle row shows the results for the landscape object layers. In both rows the confidence interval for the random patterns is shown. In the bottom row, the difference in results between the random point distribution and burrow distribution are shown, for both the square cell layers and the landscape object layers.

For all three areas, the burrow patterns (black lines) show significantly higher values than the random patterns (grey). This is the case when the values are calculated using the square cell layers (top row), as well as using landscape objects layers (middle row). This indicates that there is spatial structure in the burrow distributions in all three areas.

To evaluate the effects of using landscape objects, it is useful to compare the results from the square cell layers with the landscape object layers. For area *North*, the analysis using the landscape objects gives significantly higher values from layers with mean object sizes of ~3 km^2^ and higher. This suggests that the landscape objects better represent the structure in the burrow density compared to square cells in this area. For area *East* this is the case as for layers with mean object sizes between ~4 km^2^ and 9 km^2^. For area *West* the square cells seem to describe the data better than the landscape objects, although this is only significant for layers with a mean object size greater than ~5 km^2^.

The influence of scale on the MNABDD can be evaluated by looking at the variation of MNABDD with increasing object size. In area *West*, the difference with the mean values of the 100 random point patterns is largest at scale with a mean patch area of 3.95 km^2^. In area *North*, this difference is still increasing at 10 km^2^. In area *East*, the pattern is comparable with the pattern in area *West* and the maximum occurs at ~6 km^2^. In other words, at these scales the burrow patterns differ most from random point patterns.

### Relation burrow density and landscape-ecological variables

Linear mixed-effects models were created using the combined data of the three areas, and using all combinations of the four explanatory variables (Table 2), and their interactions.

**Table 2:** Results of the multiple regression, for the three areas combined (top row) and the areas separately

**Intercepts, coefficients and p-values multiple regression**									
	**Intercept**	**Greenness**		**Brightness**		**SRTM-SD**		**Greenness: SRTM-SD**	
		**Coeff.**	***p***	**Coeff.**	***p***	**Coeff.**	***p***	**Coeff.**	***p***
Combined	1.9	−13.3	4.7e-05	1.2	7.2e-02	0.01	0.74	2.6	0.23
*West*	1.8	−28.1	6.1-06	−1.35	0.003	0.90	0.12	9.8	0.07
*North*	1.5	−18.2	0.001	0.86	0.50	0.23	0.45	3.9	0.18
*East*	1.6	12.7	0.04	7.01	2.6e-09	−0.62	0.18	−3.2	0.49

The model with the lowest AIC (ΔAIC was >2) uses Greenness, Brightness and SRTM-SD and the interaction term Greenness:SRTM-SD (marginal R^2^ is 0.1 and conditional R^2^ is 0.2; for calculation is referred to [36]). When the spatial autocorrelation was not taken into account, all variables had a significant contribution.

The interaction term is positive, which indicates that on the floodplain (i.e. low SRTM-SD) the negative correlation of Greenness with burrow density is larger than in the dunes. The same variables were used to create models for the three separate areas (Table 2). Other models were also tested, but the models that used the same variables as for the combined model gave for all three areas the lowest AIC. Greenness shows in all areas the lowest p-values. The SRTM-SD has highest p-values, however if the interaction term was removed, SRTM-SD showed lower p-values than Brightness.

### Corridors and barriers

**Figure 9:**
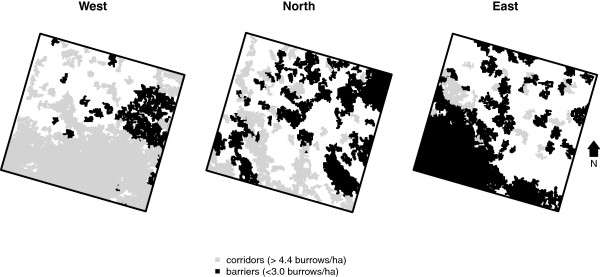
Corridors and barriers per research area.

The threshold for the functional corridors was set at mean burrow density plus one standard deviation, which equals 4.4 burrows/ha. The threshold for the functional barriers equals 3.0 burrows/ha. Maps of the functional corridors and barriers (Figure 9) show that there are differences in the amount and spatial structure of corridors and barriers between the three research areas. Most corridors occur in area *West*; most barriers occur in *East*. In area *North* and *East* the corridors and barriers seem to have a NWSE orientation.

The structural barriers (shown in blue in Figure 1) show that the Ili River forms a large barrier.

#### Spatial configuration functional corridors and barriers

**Figure 10:**
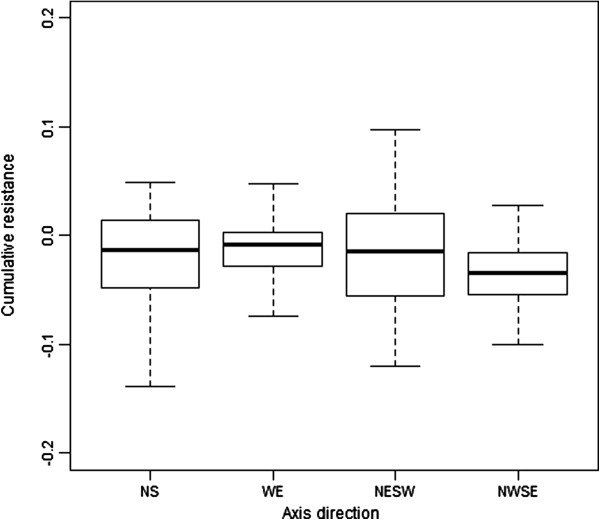
The cumulative resistance of the burrow landscape along four axes using the three study areas.

The cumulative resistance of the burrow distribution along the four axes is plotted in Figure 10. This graph shows, in theory, how easy it is for plague to spread through the landscape. In the three areas, the cumulative resistance of the burrow distribution is significantly lowest in the NWSE direction (pairwise t-test with bonferroni adjustment; p-value for NS, WE and NESW is respectively 0.0087, 6.1e-08 and 6.1e-06), or in other words, the NWSE direction offers of all directions the most suitable corridor.

### Predominant directions of plague spread

To test along which axis most plague spread occurred using the 1949–1995 plague presence/absence data, two tests were carried out: one testing whether plague spread occurred more along the NWSE + WE axes or along the NESW + NS axes, and the other whether plague spread occurred more along the NWSE + NS axes or along the NESW + WE axes (Figure 5). 1423 data points, i.e. seasons in which presence or absence of plague was recorded in one of the PSQs, were available for the former test; 1372 data points were used for the latter. The number of data points is different for the two tests, because the data points where less than two of the neighbours in each direction had been sampled for plague were omitted.

The model for directional spread of plague showed that the predominant plague spread axes are NWSE + WE (p = 0.062). The axes in which spread is occurring least are NESW + NS (Table 3). This means that in the course of 1949 to 1995 plague spread more often occurred along the NWSE and WE axis, than on the NESW and NS axes.

**Table 3:** Parameter estimates for the directional spread of plague model

	**Test 1:**		**Test 2:**	
	**NWSE + WE vs. NESW + NS**		**NWSE + NS vs NESW + WE**	
	NWSE + WE	NESW + NS	NWSE + NS	NESW + WE
*β estimates (±s.e.)*	1.83 (*±*0.5)	0.30 (*±*0.5)	1.06 (*±*0.59)	1.32 (*±*0.56)

## Discussion

In this study, the distribution of great gerbil burrows was examined in three areas located in a plague focus in Eastern Kazakhstan. Multi-scale object analysis indicated that the burrow distribution differed significantly from random. This result complies with other studies [23] and field observations suggesting that some landscapes are more suitable than others for the great gerbil. The spatial structure of the burrow pattern is explained best using the variables Greenness, Brightness, SRTM-SD and the interaction term SRTM-SD:Greenness. Higher Greenness values result in lower burrow density values, especially in the floodplain areas (i.e. when SRTM-SD is low). In the dunes (i.e. when SRTM-SD is high), Greenness has a less negative correlation with burrow density. A possible explanation for this is that great gerbils might prefer short vegetation over dense shrubs, something which has been shown for the Mongolian gerbil [37]. In the floodplain areas, high Greenness values likely correspond to the presence of tall and dense shrubs. In the dunes however, higher Greenness values correspond to dense grass cover.

The multiple regression also showed that a large part of the variation in burrow density cannot be explained by the three landscape-ecological variables. One variable that might further explain the variation in burrow density is the height of the ground water table [38]. Which other variables need to be considered is yet uncertain. Some uncertainty arises from the burrow maps themselves, as they have a mean overall accuracy of 90%. Generally, the burrow maps are more accurate in areas with little vegetation and few takirs [16]. Apart from spatial heterogeneity in the great gerbil distribution arising from heterogeneity in the landscape, it can also arise from the great gerbil’s social and behavioural interactions, or from predator-host interactions [39]. Although the great gerbil distribution in the study area was found to be non-uniform, compared to spatial distributions of other animals, the great gerbil distribution is likely relatively homogeneous. It is useful to compare spatial structures of host populations, as this can give insight in how different spatial host distributions can lead to different disease dynamics. However, few studies exist that examine the spatial structure of a population in detail. Nevertheless, some comparisons are possible. For example, prairie dogs, which are hosts of plague and live in desert areas as well, have an aggregated distribution; they live in so called “prairie dog towns”. These towns range from 0.5 to 100 ha, within which prairie dog densities also vary [40]. In between these colonies, no prairie dogs are present.

MNABDD was developed in this study, because it made it possible to evaluate the effect of using landscape objects. Another advantage of the method used here is that it possible to view the spatial pattern of the NABDD (neighbour absolute burrow density difference) within the layers, since it is calculated per object or square cell. This makes it possible to examine whether there are certain areas where the burrow differences between objects are larger than in other areas. For example, in area *North*, the largest NABDD values occur in the mid-south of the area, where there is an alternation of dunes and floodplain.

In this study, corridors and barriers were mapped in the plague focus in eastern Kazakhstan. In theory, barriers can influence disease spread and persistence in the following ways. Barriers influence the connectivity between subpopulations in a metapopulation, and hence will influence the persistence of the disease, and also the speed and direction of disease spread [41,42]. The permeability of the barriers, i.e. whether they are completely impermeable or only partially, determines the contact rates between the subpopulations on opposite sides of the barriers, and hence can influence the pattern of disease spread [42]. This makes it insightful to map barriers in a disease focus [42,43]. The structural barriers that were identified in the research area comprised canals, rivers and lakes. Although these barriers are likely not 100% impermeable to great gerbils, they do likely form a considerable barrier to plague [39]. On the other hand, disease spread can also be faster alongside rivers, which has for example been shown for rabies [44]. However, it has not been ruled out that that birds or larger mammals occasionally spread plague, in which case these structural barriers could be overcome [26].

The functional barriers were identified using the burrow distribution. These barriers were most prominent in area *East*, where there are large areas with dense shrubs present. In area *West*, the amount of barriers may be underestimated in the areas where many takirs are present. In area *North* the barriers coincide mostly with the dunes. Currently, no empirical data exist to define a burrow density threshold value. However, models such as the flea-density-threshold model [15] predict occupied burrow density threshold values below which no invasion of plague can occur. However, as the occupancy varies over space and time these modelled values cannot be translated into a single threshold burrow density value. The value we used to determine the barriers was therefore based on the statistical distribution of the burrow density data. Besides the barriers identified in this study, there may be other features acting as barriers. For example, areas where fleas cannot survive, for instance due to too high temperatures or a too low moisture content of the soil, also could act as barriers.

Apart from barriers, functional corridors using the burrow density were also mapped. No structural corridors were mapped, since no data or information existed making it possible to map them. In theory, corridors will increase the connectivity between subpopulations [45] and hence possibly influence the speed and direction of disease spread [39], and the persistence of disease [46]. In this study the functional corridors were defined as areas with high burrow densities. The underlying assumption for this is that transport of fleas between burrow systems occurs more often in high-density areas, because when distances between occupied burrows are shorter, it is more likely that fleas will be exchanged and hence plague will be transmitted. Possible other corridors for pathogen transmission could be areas where the conditions are optimal for fleas. The spatial arrangement of the corridors showed that in general the alignment of the corridors was NWSE. This was confirmed by the cost distance analysis, which showed that in all areas the NWSE direction had the highest burrow densities. An explanation for this is the geography of the landscape: the abandoned Ili River branches have a WE and NWSE direction as a result of the local topography (see Figures 1 and 2). Also the dunes are most often aligned along this axis (Figure 2), which is a result of the dominant wind directions. Both the abandoned branches as well as the dunes have lower burrow densities and hence these features are less permeable, which promotes movement along these features instead of crossing them.

The spatial arrangement of the corridors and barriers raised the question whether plague spread is radially symmetric in the plague focus, or whether there exists a predominant axis for plague spread. That the geography of a landscape and the host population structure contributes to the direction and velocity of disease spread has been shown in several studies [39,47,48]. In this study it was shown that corridors and barriers at the PSQ scale have a predominantly NWSE direction. In the PreBalkhash focus as a whole, the larger landscape features also have a predominantly NWSE direction, as Figure 1 and landscape maps of the PreBalkhash [16] show. Thus as well at a local as at a landscape scale, there are many features with a NWSE alignment. In the plague dataset, where plague spread was examined on a PSQ to PSQ scale, the predominant direction was found to be NWSE + WE. A plausible explanation for this is the NWSE structure in the spatial distribution of the burrows and the landscape. Although no detailed statistically analysed data on the direction of great gerbil movements yet exist, it has been suggested [49] that great gerbils – for example in the dunes – prefer to move from burrow to burrow on the sides of the dunes, as this is easier than crossing the dunes and also likely a safer route in terms of predation. When great gerbils are infected with plague, this would make plague spread in these directions (NW to SE and vice versa) likely. The same can apply for example to takirs that are aligned along the NWSE axis. Directional plague spread can thus be a result of gerbils moving along corridors, or in between and along barriers. It is currently yet unclear which contributes most to the directional spread of plague; for that detailed movement data of great gerbils are needed.

The plague spread model showed that spread is also more dominant along the WE axis. This is not as obviously related to the alignment of the landscape. Although some of the abandoned rivers have a WNW alignment rather than a NW alignment, overall there are less landscape features that can explain the more dominant plague spread on the WE axes. Another unanswered question is to which extent plague movement equates to gerbil movement. Plague spread is partly a stochastic process, but will depend on the behaviour of the fleas, as well as on the movement patterns of the gerbils. It is not yet clear where and when transmission of plague occurs most: is that in empty burrows visited by great gerbils from neighbouring burrows so that fleas can jump on susceptible great gerbil hosts, or does transmission occur mostly when young great gerbils migrate - along a barrier or corridor perhaps - to start a new family? Although this is yet unclear, the finding that the landscape plays a role in the spatial dynamics of plague in great gerbil populations in Central Asia underlines the value of including spatial landscape analysis in wildlife disease studies. Therefore, it is useful to include environmental variables, such as the topography, in existing plague models.

## Conclusions

In this study the landscape and burrow distribution in a plague focus in Eastern Kazakhstan were studied extensively. This was done to gain insight in the possible (in)direct influence of the landscape on the spread of plague. Plague data from 1949–1995 were used to determine whether a predominant direction of plague spread exists in this area.

The great gerbil burrow network is spatially structured and not randomly distributed. The landscape likely influences great gerbil burrow density structure. Corridors and barriers showed a NWSE alignment. This axis was also found to be predominant in the 46-year plague dataset. This makes it plausible that the landscape and the burrow network influences the direction of plague spread. This finding improves the current knowledge of the spatiotemporal dynamics of plague in Central Asia and may improve the prediction of plague in this area.

## Competing interests

The authors declare that they have no competing interests.

## Authors’ contributions

LW carried out all analyses related to the burrow data, and drafted the manuscript. LH carried out the plague data study, together with LW, and refined the regression analysis. SJ, EA and JH participated in design of the study and the manuscript. SD, AL, MB, VD and LB participated in manuscript revision. VD and LB provided useful Russian literature. All authors read and approved the final manuscript.

## Supplementary Material

Additional file 1 Supplementary information for “Potential corridors and barriers for plague spread in Central Asia”. Click here for file

Additional file 2: Figure S1 Variograms calculated based on burrow densities in the research areas *West*, *North* and *East*. Click here for file
