# Supplementary material for: Potential corridors and barriers for plague spread in central Asia
Source: Int J Health Geogr. 2013 Oct 31;12:49. doi: 10.1186/1476-072X-12-49 (PMC4228490; doi:10.1186/1476-072X-12-49)
Supplement: Additional file 1 — Supplementary information for “Potential corridors and barriers for plague spread in Central Asia”. [file 1476-072X-12-49-S1.docx]

**Supplementary information for “Potential corridors and barriers for plague spread in Central Asia”**

**Directional plague spread model**

The model for directional spread of plague (1) is an adaptation of the model used in [1]. The adapted model includes variables describing directional spread (see below) rather than one variable describing spread in unspecified directions. Further, presence/absence of plague in the model is defined by a prevalence threshold (0.005) instead of modelled presence/absence of plague. With this change, the model makes fewer prior assumptions. A threshold level of 0.005 was chosen because the overall prevalence, calculated as the total number of positive gerbils divided by the total number of gerbils tested (aggregated over all squares and all years), was 0.0058. Levels of 0.001 and 0.0004 (lowest observed positive prevalence) were used in additional analyses, to test the influence of the significance of the results.

To model directional spread of plague, we considered how the presence/absence of plague in a primary square (PSQ) is affected by plague presence in the neighbouring squares six months earlier. That is, the presence of plague in neighbouring squares in different directions was used as explanatory variable. Due to many gaps in the dataset, axes were combined two by two rather than treated separately. This means that the following plague spread axes (^ax^) were tested: NWSE + NS, NESW + NS, NWSE + WE and NESW + WE (Figure 5). Because the variables overlap, two nearly identical models were fitted to the data: one with the two variables describing NWSE + WE (*ax*1) and the perpendicular NESW + NS (*ax*2); and one with variables describing the other two axes.

The present model (1) predicts the probability that plague will be present in a given PSQ *i* at time *t* (*y_i_*_,_*_t_*=1) given four explanatory variables: the presence/absence of plague in the same square six months earlier (*y_i_*_,_*_t_*_-1_); the proportion of neighbouring squares on one of the four combined axes where plague was present six months earlier (*n^ax^*^1^*_i_*_,_*_t_*_-1_); the proportion of neighbouring squares on the perpendicular axis where plague was present six months earlier (*n^ax^*^2^*_i_*_,_*_t_*_-1_); and the great gerbil abundance in the square, averaged over the past two years (*g_t,i_*).

*β*_3_ *g_t,i_* if *y_i_*_,_*_t_*_-1_ = 0

0 if *y_i_*_,_*_t_*_-1_ = 1

logit(P(*y_i_*_,_*_t_*=1)) = *β*_0_ + *β*_1_ *y_i_*_,_*_t_*_-1_ + *β*_2,1_ *n^ax^*^1^*_i_*_,_*_t_*_-1_ + *β*_2,2_ *n^ax^*^2^*_i_*_,_*_t_*_-1_ +

(1)

The variables describing directional spread, here called presence/absence of plague in neighbouring squares, were calculated as the number of squares with plague, divided by the total number of neighbouring squares in the relevant directions where sampling for plague had been performed. The condition was made that at least two of the four neighbouring squares in the relevant directions must be sampled if the data point was to be included in the analysis.

To test the null hypothesis that spread is equally important in two perpendicular directions, *β*_2,1_ = *β*_2,2_, the model was reparameterized so that one variable described spread in unspecified directions (*n^ax^*^1^ + *n^ax^*^2^) and one described the difference in spread in perpendicular directions (*n^ax^*^1^ – *n^ax^*^2^).

Uncertainty in plague data due to small sample sizes was dealt with by a series of imputations of plague data, by use of a bayesian technique. The procedure for imputation and analysis is given below. The statistical software R [2]was used, with the methods glmmPQL within the MASS library [3] and MIcombine within the mitools package [4]. For further details, see [1].

1. The prior assumption is that the prevalence is beta-distributed with parameters *a* and *b*. The values *a* = 0.0058 and *b* = 1 were used, giving a relatively uninformative prior with a mean of 0.0058 (corresponding to the mean prevalence when all data are pooled together). The results are relatively robust to changes in *a* and *b*.
2. For each square, given the number of great gerbils tested (*r_t_*) and the number of great gerbils found positive (*r_p_*), the posterior probability α that prevalence > 0.005 was then given by the beta-distribution with parameters *a* + *r_p_* and *b* + *r_t_* – r*_p_*.
3. By drawing from a Bernoulli distribution with probability α, 50 imputed binary datasets representing prevalence of plague > 0.005 were made.
4. For each imputed dataset, a logistic mixed model was fitted by using the R method glmmPQL. The fixed effects are described by model (1). For the spatial correlation in the binary data, an exponential correlation structure with nugget effect was used.
5. The 50 models were combined by the method MIcombine in R to yield summary estimates and standard errors.

These imputations were only performed in squares where the number of tests performed, *r_t_*, was positive (for more information on this procedure see [1]).

**Model results**

The estimates of the parameters in model (1) are given in table S.1 (fixed effects only). The difference in directional spread between NWSE + WE and NESW + NS is nearly significant with a p-value of 0.062 when the presence/absence threshold is set to 0.005. A threshold of 0.001 resulted in p = 0.085, while a further reduction to 0.0004 resulted in p = 0.021.

**References**

1. Heier L, Storvik GO, Davis SA, Viljugrein H, Ageyev VS, Klassovskaya E, Stenseth NC: **Emergence, spread, persistence and fade-out of sylvatic plague in Kazakhstan.** Proceedings of the Royal Society B: Biological Sciences; 2011.

2. R Development Core Team: *R: A Language and Environment for Statistical Computing:* Vienna, Austria: R Foundation for Statistical Computing; 2011.

3. Venables WN, Ripley BD: *Modern applied statistics with S  (Statistics and Computing):* 4th ed. New York: Springer; 2002.

4. Lumley T: ***mitools*: Tools for multiple imputation of missing data. R package version 2.0.1**; 2010.

**Tables**

**Table S.1 Parameter estimates in the model for directional spread of plague. The underlined p-value shows that the axis NWSE+ WE is nearly significant.**

|  | Estimate | Std. Error | Pr(>\|t\|) |
| --- | --- | --- | --- |
| NWSE + WE vs. NESW + NS |  |  |  |
| *β*_0_ | -5.33 | 0.55 | <2.0e-16 |
| *β*_1_ | 3.59 | 0.61 | 7.45e-09 |
| ½(*β*_2,1_ + *β*_2,2_) | 1.06 | 0.32 | 1.10e-03 |
| ½(*β*_2,1_ - *β*_2,2_) | 0.77 | 0.41 | 6.23e-02 |
| *β*_3_ | 0.04 | 0.01 | 1.67e-06 |
|  |  |  |  |
| NESW + WE vs. NWSE + NS |  |  |  |
| *β*_0_ | -5.27 | 0.56 | <2.0e-16 |
| *β*_1_ | 3.68 | 0.63 | 9.20e-09 |
| ½(*β*_2,1_ + *β*_2,2_) | 1.15 | 0.34 | 8.23e-04 |
| ½(*β*_2,1_ - *β*_2,2_) | 0.13 | 0.43 | 7.57e-01 |
| *β*_3_ | 0.04 | 0.01 | 2.99e-06 |
