# Supplementary figures and images for: Potential corridors and barriers for plague spread in central Asia
Source: Int J Health Geogr. 2013 Oct 31;12:49. doi: 10.1186/1476-072X-12-49 (PMC4228490; doi:10.1186/1476-072X-12-49)

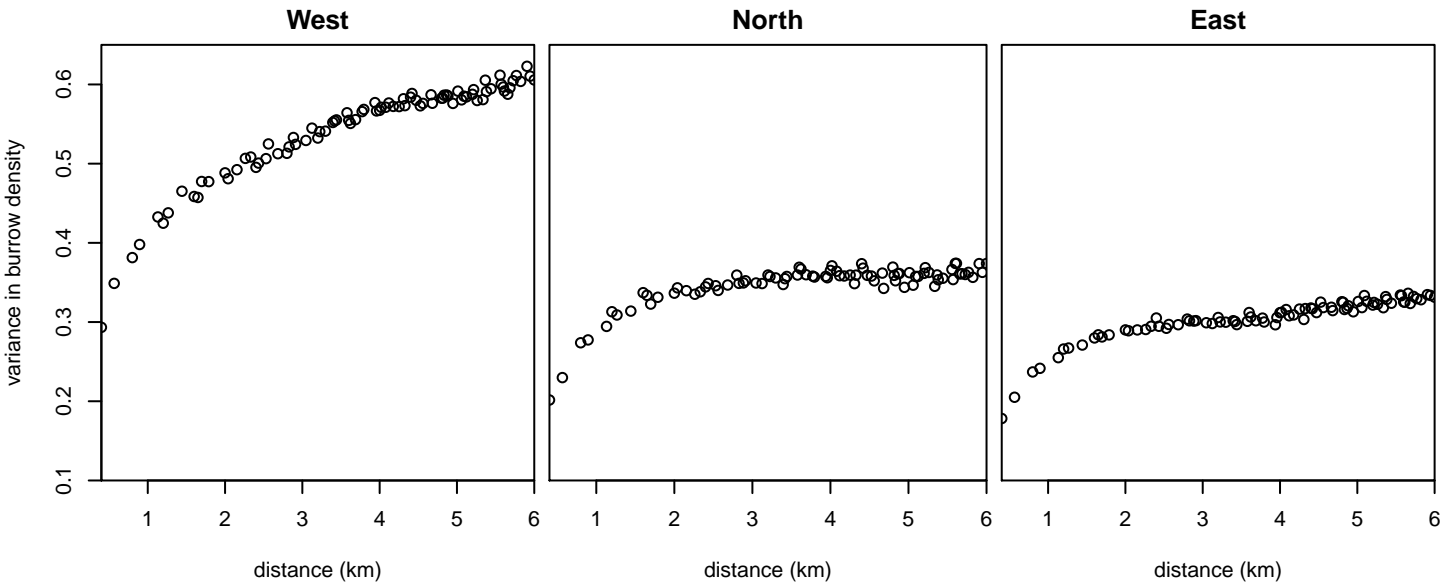

Supplement: Additional file 2: Figure S1 — Variograms calculated based on burrow densities in the research areas West, North and East. [file 1476-072X-12-49-S2.pdf]
